# A lung cancer patient with deep vein thrombosis:a case report and literature review

**DOI:** 10.1186/s12885-019-5513-8

**Published:** 2019-03-29

**Authors:** Yungu Chen, Yiu Sing Tsang, Xiaoxia Chou, Jiong Hu, Qing Xia

**Affiliations:** 1 Department of Basic Medical Sciences, Shanghai Jiao Tong University School of Medicine, Shanghai, 200025 China; 2 Department of Oncology, Affiliated Renji Hospital, Shanghai Jiao Tong University School of Medicine, Pancreatic Cancer Center of Shanghai Jiao Tong University, Shanghai, 200127 China

**Keywords:** Case report, Deep venous thrombosis, Lung cancer, Chemotherapy

## Abstract

**Background:**

Venous thromboembolism (VTE) is a common problem in cancer patients and the incidence is increasing, especially for patients with lung cancer. Common features of these patients, like advanced stage, male gender, old age and chemotherapy, are risk factors of VTE. Here we reported a case in which the patient with lung cancer developed deep vein thrombosis (DVT) when receiving chemotherapy.

**Case presentation:**

A 53-year-old male who was diagnosed with lung cancer with multiple metastasis developed severe DVT during chemotherapy. Despite the use of aspirin, warfarin and low molecular weight heparin (LMWH) for anticoagulant and thrombolytic therapy, the condition was still deteriorating, resulting in amputation finally.

**Conclusions:**

It’s rare that the conditions of cancer patients who develop venous thromboembolism (VTE) keep deteriorating despite the administration of aspirin, warfarin and low weight molecular heparin. Both early diagnosis and prophylactic use of anticoagulants are suggested for cancer patients to improve the prognosis.

## Background

Venous thromboembolism (VTE), a thrombotic disorder defined as deep vein thrombosis (DVT), pulmonary embolism (PE), or both, is a clinical challenge in patients with malignancies, which has caused unneglectable morbidity and mortality.

The risk of VTE for cancer patients is four to seven times higher than patients free from cancer, and around 15% of cancer patients are affected with a VTE episode [1]. Cancer patients also have an increasing risk of developing VTE during the entire course of their illness. Nevertheless, after the commencement of the treatment for cancer such a risk is even higher, especially during the first few months after diagnosis and at the end-stage of cancer [2].

Among all cancer-related mortality, lung cancer is still the leading cause worldwide, with tobacco intake as the main known risk factor. More than 85% of lung cancer diagnoses are attributable to tobacco smoking and a large proportion (40%) of patients are active smokers at the time of diagnosis [3]. Meanwhile, smoking is a risk factor of developing cardiovascular disorders as well and hence may plausibly be the cause of VTE in terms of a broader spectrum. Despite a known correlation between VTE and lung cancer, a comprehensive guideline of the prophylaxis and treatment for patients with lung cancer who develop VTE is still needed due to the limited number of studies conducted on the matter.

In this case report, we discussed clinical manifestations, risk factors of VTE and treatment procedures in a patient with advanced-stage lung cancer who developed severe DVT and eventually underwent amputation. Additionally, we reviewed and discussed current guidelines for cancer patients with VTE to find ways that might improve prognostic outcomes.

## Case presentation

A 53-year-old male was hospitalised because of pain in the right hip that was ongoing for 3 months. On physical examination, positive signs included the enlargement of his right supraclavicular lymph node of 0.5 cm * 0.5 cm, and overt tenderness in his right groin with no mass palpated. The patient had a symmetric chest and breathing sounds of both lungs were clear on auscultation and no rales or crackles were heard. The abdomen was soft without any tenderness of rebound pain, bowel sounds were normal, and there was no redness or swelling found in any limbs.

Blood routine, liver and kidney function, coagulation function for the patient were normal, except that the fibrinogen was 4.68 g/l, slight above average (Ref int 2.00–4.00 g/l).

**Fig. 1:**
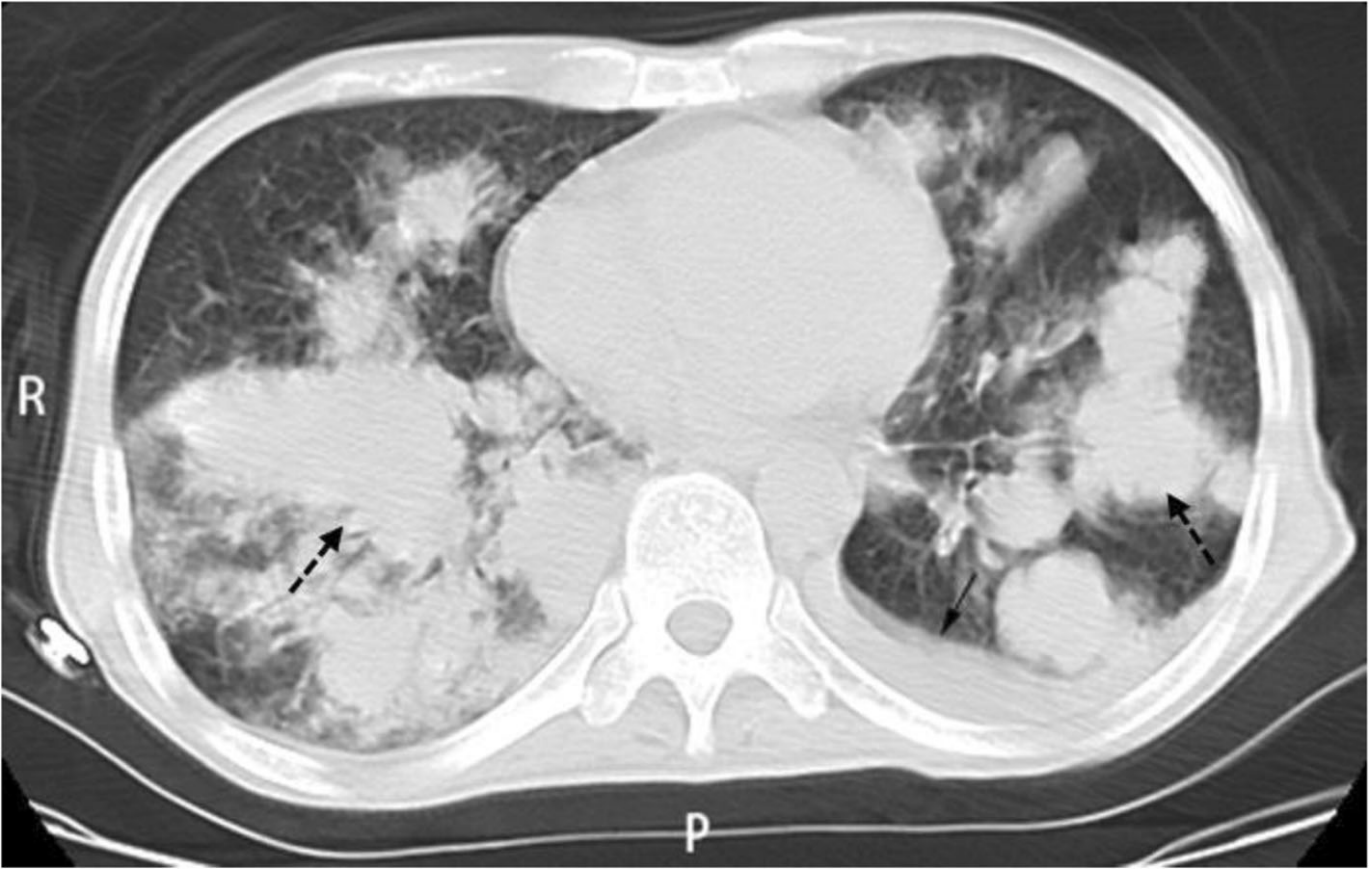
PET-CT indicated lung cancer with dissemination to both lungs (dashed arrows) and pleural effusion (solid arrow) on the left side

**Fig. 2:**
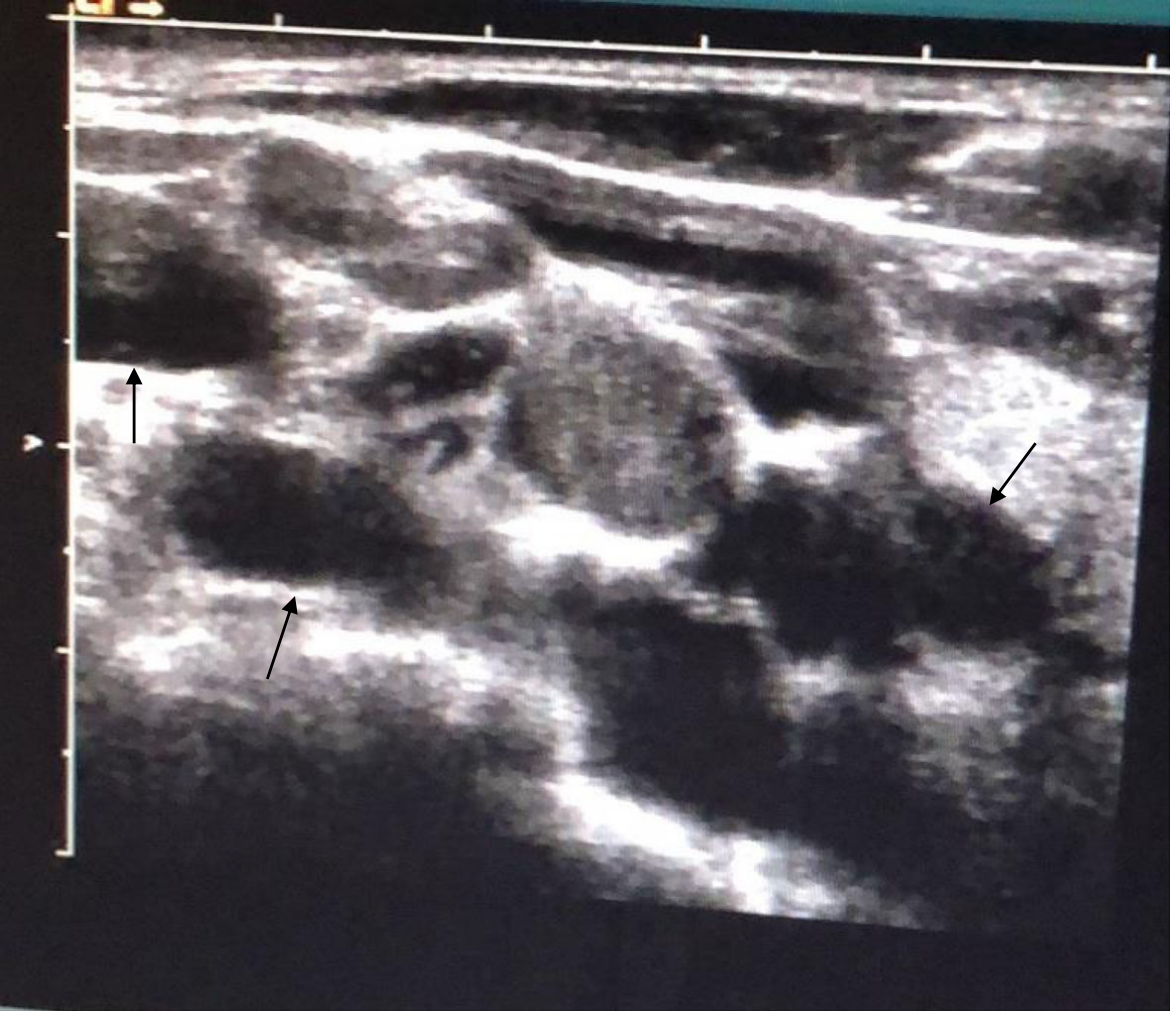
Ultrasound indicated multiple lymph node enlargement in bilateral supraclavicular regions (solid arrows)

Computed tomography (CT) indicated malignant lesions of left upper lobe and multiple lymph node metastases in mediastinum, neck, both lungs and right acetabular bone (Fig. 1). Fluorodeoxyglucose (FDG) metabolism of the left adrenal gland was slightly elevated. Multiple cysts were noticed in the liver and the left kidney. Ultrasound indicated multiple lymph node enlargement in bilateral supraclavicular regions (Fig. 2).

Past history revealed the patient had a smoking history for 30 pack years but was otherwise unremarkable. The patient denied any history of tuberculosis infection or thrombotic events. The level of tumor markers is shown in Table 1.

**Table 1:** Tumor markers of the patient

Description	Result	Unit	Ref int
CEA	14.60↑	ng/ml	0—5
CA199	3.10	U/ml	0—35.00
NSE	33.03	ng/ml	0–16.3

*NSE*: Neuron-specific enolase; *CEA*: Carcinoembryonic antigen; *CA199*: Carbohydrate antigen 199, *Ref int*: Reference interval

The right supraclavicular lymph node was surrounded by vessels, making the biopsy impossible. The patient’s family refused needle biopsy of lung lesions. The clinical diagnosis of multiple metastasis of lung cancer was then made.

After the diagnosis, the patient received palliative radiotherapy 2.5Gy/Fx on metastasis at the right acetabular bone, combined with zoledronic acid treatment and pain management. 5 days later, central catheters were inserted peripherally and chemotherapy (Taxol 210 mg dl + cisplatin 40 mg dl-3 q21d) was administered.

8 days after the initiation of chemotherapy, the right hip pain was alleviated. 2 days later, the patient described he felt distending pain in the left lower limb. Coagulation lab tests were as follows.

**Fig. 3:**
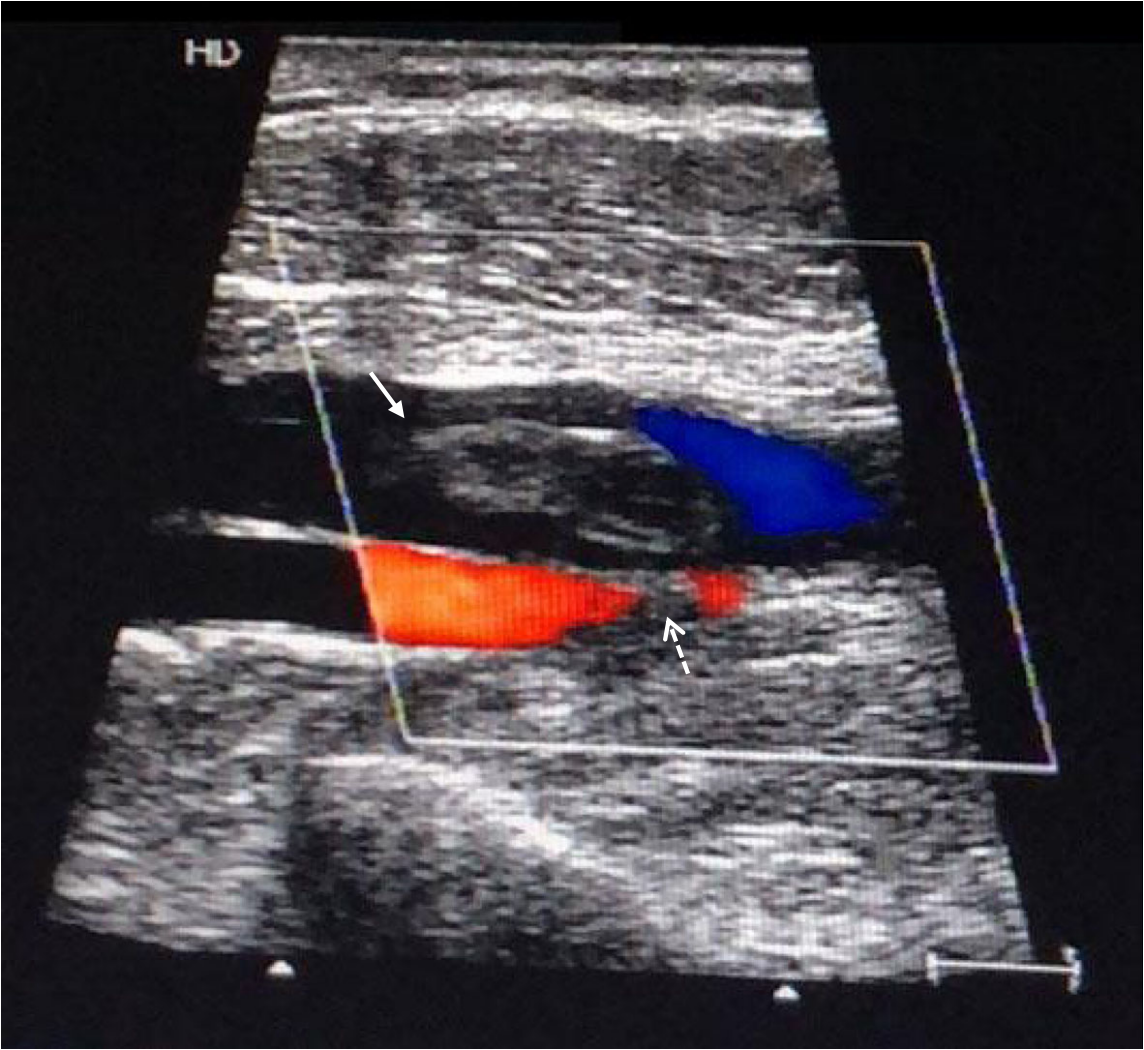
Venous duplex ultrasonography indicated absence of blood flow in the thrombosed left femoral vein (solid arrow) and compression of the left femoral artery (dashed arrow). The diameter of the vein examined was still considerably broad when compressed

Venous duplex ultrasonography indicated deep vein thrombosis (DVT) of the left femoral vein by detecting noncompressibility of the thrombosed vein with reduced blood flow. Compression of the left femoral artery caused by thrombosed vein was also noticed (Fig. 3).

Anticoagulant therapy (Aspirin) and thrombolytic therapy (Warfarin and low molecular weight heparin (LMWH)) were administered. However, the symptoms were progressively deteriorating. 4 days later, the distending pain had worsened, and new symptoms appeared including cyanosis and lower skin temperature. Ultrasound and CT-angiography (CTA) indicated insufficient blood supply to the left lower limb due to compression on arteries by DVT. The attempt of embolectomy and inferior vena cava (IVC) filter implantation was unsuccessful. The post-surgery biopsy indicated the thrombosis was a blood clot. Because the stent failed to enter the left superficial femoral vein where multiple older thrombi existed. The symptoms of DVT was deteriorating, with gangrene arising in the left lower limb. Thus, amputation of the left thigh was eventually performed.

## Discussion and conclusions

DVT is a well-studied cancer complication and the incidence is estimated to be from 4 to 17% in patients presenting underlying malignancies [4]. Cancer patients are predisposed to the development of VTE events with an up to 7% risk, partly due to their considerable exposure to various circumstantial risk factors including surgery, immobilization and medications during the course of their disease. Additionally, cancer is often associated with a prothrombotic state, which might be clinically asymptomatic or lead to VTE that is resistant to anticoagulants [5]. When compared to cancer patients without thrombosis, patients with both thrombotic events and cancer have a lower survival rate. Furthermore, patients with malignancies and concomitant VTE have an increased complication rate from anticoagulation therapy and increased risk of a second VTE episode [6].

It’s recommended that the risk of VTE should be assessed before the commencement of chemotherapy [7]. Efforts have been made to recognize the predictors of the VTE episode in lung cancer patients and several risk models were established to assign patients to risk classes. Khorana risk score (KRS) is a validated prediction score for cancer-associated thrombosis. The biomarkers concerned in KRS include the site of cancer, platelet counts, leukocyte counts, haemoglobin and body mass index (BMI) [8] (Table 2). Nonetheless, in a review of 719 patients, a high KRS was not associated with VTE compared to an intermediate score in both univariate and multivariable analyses. A high KRS, however, was a mortality predictor [9]. The patient in this case had a KRS of 1, placing him in an intermediate-risk group. Protecht score is an improved risk assessment method where platinum or gemcitabine-based chemotherapy was added to the predictive variables that are already taken into account in the Khorana score. Compared to the Khorana score, Protecht score demonstrated a bettered ability to identify cancer patients who are at high risk for VTE [10]. Based on the original Knorana score, Vienna prediction score added P-selectin and D-dimer, two markers of platelet aggregation and clotting cascade respectively, which considerably improved prediction of VTE [11] (Table 3).

**Table 2:** Coagulation lab results of the patient

Description	Result	Unit	Ref int
D-dimers	27.53↑	ug/ml	0.0–0.5
TT	15.2	S	10.3–16.6
PT	14.2↑	S	9.8–12.8
Fibrinogen	2.92	g/l	2.00–4.00
APTT	32.9	S	25.1–36.5
INR	1.17↑	R	0.8–1.15

*TT*: Thrombin time; *PT*: Prothrombin time; *APTT*: Activated partial thromboplastin time; *INR*: International normalized ratio, *Ref int*: Reference interval

**Table 3:** Khorana risk score [8]

Patient characteristic	Score
Site of cancer	
Very high risk (stomach, pancreas)	2
High risk (lung, lymphoma, gynecologic, genitourinary excluding prostate)	1
Platelet counts ≥350,000 per mm^3^	1
Leukocyte counts > 11,000 per mm^3^	1
Hemoglobin < 10 g/dL or use of ESAs	1
BMI ≥35 kg/m^2^	1

High-risk score ≥ 3; Intermediate risk score = 1–2; Low-risk score = 0. Primary brain tumor and myeloma patients were not part of this study. Information on the impact of prior VTE is also not available in this study. *BMI*: Body mass index; *ESA*: Erythropoiesis-stimulating agent

Apart from the biomarkers mentioned in the above risk-evaluation models, it is found that some other individual biomarkers are also correlated with the development of VTE in lung cancer patients. For example, besides surgical interventions and hospitalization, chemotherapy with cisplatin [12], immunomodulatory drugs (thalidomide analogues) or angiogenic inhibitors increases risk for thrombosis [6]. Advanced stage is confirmed to be a strong predictor of VTE in patients with non-small cell lung carcinoma (NSCLC). It is observed in several studies that there is a direct association between the cancer stage and thrombosis risk, patients with stage III or IV NSCLC being more predisposed to VTE [4, 13]. The tumor grade may help identify patients with cancer who are at high risk of VTE [14]. Yang W et al. demonstrated that platelet-lymphocyte ratio (PLR) at the time of cancer diagnosis could be a useful clinically important, independent risk predictor for VTE in cancer patients, where PLR > 260 could predict a VTE episode [15].

Guidelines regarding the use of prophylactic anticoagulants in lung cancer patients receiving chemotherapy varies. The American College of Chest Physicians (ACCP) does not recommend the use of thromboprophylaxis [16]; The European Society for Medical Oncology (ESMO) recommends that in cases of VTE high-risk patients with solid tumors are considered case by case and that the decision be discussed with patients [17]; the American Society of Clinical Oncology (ASCO) guidelines recommend all hospitalized cancer patients should be considered for VTE prophylaxis with anticoagulants in the absence of bleeding or other contraindications [18]; the International Society on Thrombosis and Haemostasis (ISTH) recommends the use of thromboprophylaxis in patients with advanced/metastatic cancer of pancreas or lung at low risk of bleeding [19]. There is still a long way to go between guidelines and clinical practice and consensus is needed.

Some factors might reduce the effect of treatment. The lung cancer in this patient was in the late phase, spreading throughout both lungs. Failure to notice the thrombotic event early caused delayed treatment. Additionally, the patient also had many risk factors predisposing VTE, like smoking, bed rest and high coagulability caused by the malignancy. All these factors might explain why treatment did not work. Differential diagnosis includes heparin-induced thrombocytopenia (HIT), Antiphospholipid Syndrome and warfarin necrosis. For HIT to be diagnosed, patients with proven thrombosis must present with a significant fall in platelet 5–10 days after the commencement of heparin treatment [20]. In this case, however, the patient didn’t show a significant fall in platelet, making the diagnosis of HIT less likely. The diagnosis of Antiphospholipid Syndrome, an autoimmune disease, requires the detection of autoantibodies. Although lab tests concerning this disease were not ordered by corresponding physicians, a lack of any history of thrombotic events or autoimmune defects makes Antiphospholipid Syndrome a less likely etiology. The risk of warfarin necrosis is normally reduced by concomitantly administrated heparin, because heparin takes a different mechanism than warfarin.

The anticoagulant therapy was not initiated until the patient developed distending pain of the left lower limb, with venous duplex ultrasonography confirming DVT. Aspirin, warfarin and LMWH were used as the initial treatment in this case. ACCP recommends the use of heparin and vitamin K antagonist to treat patients who are highly suspected of acute DVT while waiting for the results of diagnostic tests. [21] Aspirin was used empirically as an anti-platelet agent. Despite the use of anticoagulants, the outcome was very poor, eventually leading to the last choice of amputation when gangrene developed. Although there are diverse results in the statistical significance in the use of prophylactic anticoagulants [22, 23], the low complication rates observed with prophylaxis in the major medical trials appear to justify the use of pharmacologic prophylaxis in hospitalized patients with cancer. A better outcome might be had if the anticoagulant therapy were started from the beginning of chemotherapy instead of the moment when DVT symptoms developed.

In conclusion, cancer patients, especially those whose cancer originates in the lung, are predisposed to the development of DVT. Khorana score, Protecht score and Vienna score could be considered in the evaluation of the risk of the VTE episode. Advanced stage, surgery, immobility, chemotherapy and PLR are important predictors for these patients. A prophylactic use of anticoagulants is recommended for patients with advanced lung cancer.

## Abbreviations

BMI: body mass index
CA199: carbohydrate antigen 199
CEA: carcinoembryonic antigen
CT: computed tomography
DVT: deep vein thrombosis
FDG: fluorodeoxyglucose
IVC: inferior vena cava
KRS: Khorana risk score
LMWH: low molecular weight heparin
NSCLC: non-small cell lung carcinoma
NSE: neuron-specific enolase
PE: pulmonary embolism
PLR: platelet-lymphocyte ratio
VTE: venous thromboembolism
